# Feasibility, safety, efficacy and potential scaling-up of sofosbuvir-based HCV treatment in Central and West Africa: (TAC ANRS 12311 trial)

**DOI:** 10.1038/s41598-024-57013-1

**Published:** 2024-05-03

**Authors:** Karine Lacombe, Raoul Moh, Corine Chazallon, Maud Lemoine, Babacar Sylla, Fatoumata Fadiga, Jerôme Le Carrou, Fabienne Marcellin, Charles Kouanfack, Laura Ciaffi, Michelle Tagni Sartre, Magloire Biwole Sida, Alpha Diallo, Joel Gozlan, Moussa Seydi, Viviane Cissé, Christine Danel, Pierre Marie Girard, Thomas d’Aquin Toni, Albert Minga, Sylvie Boyer, Patrizia Carrieri, Alain Attia, The TAC ANRS12311 Study Group, Sophie Karcher, Pierre Touret, Camara Mory, Laté Mawuli Lawson-Ananissoh, Romuald Konan, Ndèye Aissatou Lakhe, Batsy Koita Fall, Bara N’Diaye, Coumba Toure Kane, Michelle Tagni-Sartre, Isabelle Dang Babagna, Eric Pascal Tchoumi, Eitel Mpoundi Ngole, Avelin Aghokeng, Rina Djubgang

**Affiliations:** 1 Infectious Diseases Department, Inserm IPLESP, UMR-S1136, Hôpital Saint-Antoine, AP-HP, Sorbonne Université, Paris, France; 2 Unité Pédagogique de Dermatologie et Infectiologie, Université Félix Houphouet-Boigny, Abidjan, Côte d’Ivoire; 3 Programme PAC-CI, Site ANRS de Côte d’Ivoire, Abidjan, Côte d’Ivoire; 4 National Institute for Health and Medical Research (INSERM) UMR 1219, Research Institute for Sustainable Development (IRD) EMR 271, Bordeaux Population Health Centre, University of Bordeaux, Bordeaux, France; 5 Hepatology Unit, Digestive Disease Division, Imperial College London, St Mary’s Hospital, London, UK; 6 IMEA, Hôpital Bichat – Claude Bernard, Paris, France; 7 Inserm, IRD, SESSTIM, Sciences Economiques & Sociales de la Santé & Traitement de l’Information Médicale, ISSPAM, Aix Marseille Univ, Marseille, France; 8 Hôpital de Jour, Hôpital Central, Yaoundé, Cameroon; 9 TransVIHMI – IRD UMI233 – INSERM U1175, Université de Montpellier, Montpellier, France; 10 Clinique de la Cathédrale, Yaoundé, Cameroon; 11 Faculté de Médecine et des Sciences Biomédicales, Université de Yaoundé 1, Yaoundé, Cameroon; 12 Service de Pharmacovigilance, ANRS, Paris, France; 13 Department of Virology, INSERM, UMR_S 938, Centre de Recherche Saint-Antoine, Hôpital Saint-Antoine, AP-HP, Sorbonne Université, Paris, France; 14 Service des Maladies Infectieuses et Tropicales, CHNU de Fann, Dakar, Senegal; 15 Service des Maladies Infectieuses et Tropicales, Centre Régional de Recherche et de Formation, Site ANRS, CHNU de Fann, Dakar, Senegal; 16 Service de Virologie, Centre de diagnostic et de recherche sur le SIDA, CHU Treichville, Abidjan, Côte d’Ivoire; 17 Centre National des Donneurs de Sang, Abidjan, Côte d’Ivoire; 18 Service d’hépatologie, CHU de Yopougon, Abidjan, Côte d’Ivoire; 19 Service des Maladies Infectieuses et Tropicales, Hôpital St Antoine, 184 rue du Fbg St Antoine, 75012 Paris, France; 20 Université Cheikh Anta Diop, Dakar, Senegal

**Keywords:** Hepatitis C, HIV, Africa, Sofosvubir, Clinical trial, Viral resistance, Gastroenterology, Medical research

## Abstract

Access to Hepatis C treatment in Sub-Saharan Africa is a clinical, public health and ethical concern. The multi-country open-label trial TAC ANRS 12311 allowed assessing the feasibility, safety, efficacy of a specific care model of HCV treatment and retreatment in patients with hepatitis C in Sub Saharan Africa. Between November 2015 and March 2017, with follow-up until mid 2019, treatment-naïve patients with HCV without decompensated cirrhosis or liver cancer were recruited to receive 12 week-treatment with either sofosbuvir + ribavirin (HCV genotype 2) or sofosbuvir + ledipasvir (genotype 1 or 4) and retreatment with sofosbuvir + velpatasvir + voxilaprevir in case of virological failure. The primary outcome was sustained virological response at 12 weeks after end of treatment (SVR12). Secondary outcomes included treatment adherence, safety and SVR12 in patients who were retreated due to non-response to first-line treatment. The model of care relied on both viral load assessment and educational sessions to increase patient awareness, adherence and health literacy. The study recruited 120 participants, 36 HIV-co-infected, and 14 cirrhotic. Only one patient discontinued treatment because of return to home country. Neither death nor severe adverse event occurred. SVR12 was reached in 107 patients (89%): (90%) in genotype 1 or 2, and 88% in GT-4. All retreated patients (n = 13) reached SVR12. HCV treatment is highly acceptable, safe and effective under this model of care. Implementation research is now needed to scale up point-of-care HCV testing and SVR assessment, along with community involvement in patient education, to achieve HCV elimination in Sub-Saharan Africa.

## Introduction

In 2021, the World Health Organization (WHO) estimated 58 million hepatitis C cases worldwide and about 1.5 million incident cases annually; in 2019, 290,000 people died from hepatitis C, mostly from cirrhosis and hepatocellular carcinoma (HCC) (primary liver cancer)^1^. Africa is endemic for hepatitis C virus (HCV) infection, which is the second leading cause of end-stage liver disease and HCC across the continent. In Sub-Saharan Africa (SSA), reported HCV prevalence has reached nearly 3%^2^, but great geographical variations are observed with estimates up to 4–6% in West and Central Africa^3^.

In its recent global hepatitis elimination plan, the WHO set ambitious targets, defined by a 90% reduction in new HCV cases and a 65% decrease in HCV-related mortality. To achieve these targets, a substantial increase in HCV-treatment coverage of at least 80% by 2030 is needed^4^. Following the availability of direct-acting antiviral-agents (DAAs) now accessible at generic costs in most low-to-middle-income countries (LMICs), HCV elimination has become an attainable objective for many countries. A mathematical model showed that providing DAAs at the time of chronic hepatitis C (CHC) diagnosis in all countries could avert 640,000 deaths from HCV-related cirrhosis and HCC^5^. However, multiple barriers (e.g., inadequate national hepatitis programmes, resource-limited health care systems and poor awareness and stigmatisation) undermine HCV testing and treatment, especially in LMICs^6^. Egypt^7^ and Rwanda^8^ have deployed remarkable efforts to develop HCV elimination strategies. Both countries have implemented national hepatitis C test-and-treat interventions, which have informed national hepatitis elimination plans^9,10^. Recently, some consensus has been reached about HCV guidelines adapted to the African setting^11^ despite knowledge gaps on DAA efficacy in real-life settings and the lack of implementation models of HCV care.

Although the prevalence of specific and rare HCV genotypes may affect the response rate in SSA, this is not a solid argument to delay care in this region, especially given the results of studies conducted in France, which showed that migrants from SSA^12,13^ obtained comparable sustained virological response (SVR) rates as other groups. However, the lack of free access to HCV care, the unavailability of HCV care at decentralized level of the healthcare system and the social context (e.g., stigma exerted against people with HCV^14^) may affect the continuity and response to HCV treatment. Undeniably, by 2040, global mortality related to viral hepatitis will exceed the overall number of deaths from HIV, tuberculosis and malaria^15^. Most of these deaths will occur in LMICs, the region of the world most affected by viral hepatitis, unless hepatitis B and C policy and implementation research help draw the road map for hepatitis elimination in these countries.

To date, the main question remains to what extent treatment of HCV is feasible and effective in LMICs. Though simplified models of care have already shown their effectiveness in some LMIC settings^8^, there are limited data on the effectiveness of DAAs, overall and in case of retreatment due to virological failure. Moreover, conducting operational research and implementation science can offer the opportunity to build a network of clinicians, virologists and researchers across several European and African countries in the field of HCV.

We conducted the TAC-ANRS 12311 clinical multi-country trial to assess the feasibility, safety and efficacy of a sofosbuvir (SOF)-based therapy in treatment-naïve subjects with CHC and of retreatment of failing patients. The study was conducted in Cameroon, Côte d’Ivoire and Senegal, where HCV sero-prevalence is estimated at 4.9%, 2.2% and 1%, respectively^16^. We adopted a model of care including scheduled educational sessions to increase patient awareness, adherence and health literacy and a dedicated network of staff and community members.

## Methods

### Study design

The TAC-ANRS 12311 trial is an international, multicenter, open-label, non-comparative, non-randomised, phase 2b clinical trial designed to evaluate a 12-week SOF-based regimen combined with ribavirin (RBV) (HCV genotype 2) or ledispavir (HCV genotypes 1 and 4) for adults with CHC who are either mono-infected or HIV co-infected in low-income countries. Participants were enrolled between 9 November 2015 and 1 March 2017 in four infectious disease departments of hospitals or clinics located in the study countries’ capital cities (Hôpital Central and Clinique de la Cathédrale, Yaoundé, Cameroon; Hôpital Fann, Dakar, Senegal; CHU de Yopougon and Centre Médical de Suivi des Donneurs de Sang, Abidjan, Côte d’Ivoire).

### Eligibility criteria

Eligibility criteria were as follows: age ≥ 18; chronic HCV infection proven by positive HCV serology (3rd generation ELISA IgG test) and an HCV-RNA ≥ 12 UI/mL, proven GT-1, -2, or -4, no previous exposure to anti-HCV drugs; agreement of women to use contraception one-month prior to treatment initiation and four months after treatment completion and for men, use of condoms, started at least 1 month prior treatment initiation and 7 months after treatment completion; weight between 40 and 125 kg; and signed informed consent. In addition, HIV-positive patients should fulfill the following criteria: having a confirmed HIV infection (3rd generation ELISA and Western blot), receiving stable antiretroviral treatment containing drugs compatible with the DAAs used in the trial, having CD4 lymphocyte count > 100 cells/mm^3^ and HIV-RNA < 200 copies/mL.

Non-inclusion criteria were having a positive HBsAg rapid test, known Child–Pugh B or C cirrhosis, being pregnant or lactating women, history of organ transplantation, comorbidity such as cancer, epilepsy, drepanocytosis, myocardial infarction, QT elongation ≥ 20 ms, alcohol consumption above 20 g for women or 40 g for men, active drug use, haemoglobin < 10 g/mL in men and 11 g/mL in women, platelets count < 50,000/mm^3^, neutrophils count < 750/mm^3^, creatinine clearance with MDRD (Modification of Diet in Renal Disease) formula < 50 mL/Min, history of opportunistic infections in the previous 6 months and history of non-adherence to antiretroviral treatment for HIV co-infected patients.

### Treatment

Eligible patients with HCV genotype (GT)-1 or -4 received SOF 400mg combined with ledipasvir (LDV) 90mg one pill per day for 12 weeks, whereas patients with GT-2 received SOF 400 mg one pill per day with RBV 200 mg, 5 pills/day if weight < 75 kg or 6 pills/day if weight ≥ 75 kg for 12 weeks. The choice of DAA type was driven by the 2015 International Guidelines^17^, valid at the time of study design. Virological failure was defined as a detectable HCV-RNA 12 weeks after end of treatment (EOT). Patients failing first-line DAA were retreated using SOF 400mg/velpatasvir (VEL) 100 mg/voxilaprevir (VOX) 100 mg one pill a day for 12 weeks.

## Model of care

The model of care was built upon two primary components: viral load monitoring and patient education upon treatment initiation. Scheduled patient education sessions, lasting approximately 15–20 min, were conducted at treatment initiation, as well as during weeks 2, 4, 8, and 12. This educational content aimed to enhance understanding of the disease, address the challenges associated with achieving HCV cure, underscore the importance of treatment adherence, and provide guidance on managing potential side effects, emphasizing the treatment’s low toxicity. Additionally, the week 4 session provided an opportunity for patients to share their treatment experiences and discuss any adherence difficulties they may have encountered.

### Biological and clinical assessments

Participants were followed for 48 weeks. Clinical evaluation and laboratory monitoring (including renal and liver function test, total blood count and HCV-RNA quantification) were performed at Week 2, 4, 8, 12 and 24. Additional biological tests (including bilirubin, albumin, prothrombin time, alpha-fetoprotein as well as CD4 cell count and HIV viral load for HIV-infected participants) were also performed at Week 24, i.e., 12 weeks after EOT. Participants with a positive HCV-RNA 12 weeks after EOT were considered as failing treatment. Treatment failure was also reassessed at Week 36, i.e., 24 weeks after treatment end, to identify potential late treatment failure, which might occur in participants initially in treatment success at Week 24.

HCV-RNA quantification was performed using automatized real time PCR, either with Roche AmpliPrep/COBAS® TaqMan® HCV Quantitative Test assay, or Abbott Real time M2000 SP/M2000RT PCR assays, with a 12 UI/mL limit of detection. When HCV-RNA was detected, genotypic sequencing was performed using an NS5B in-house genotyping test.

Retreated patients also received information about the crucial role of adherence to this second-line treatment, the absence of alternative treatment in case of further virological failure and the management of possible side effects.

Liver fibrosis was assessed with the APRI combining the AST rate and platelets count, according to the current WHO guidelines at the time the trial was designed. The threshold of 2 has been retained to diagnose cirrhosis^18^. APRI has been assessed at each follow-up visit, i.e. pre-treatment then week 2, 4, 8 and 12, as well as at the end of treatment.

All other biological tests were performed using local laboratory infrastructures; all of them were subject to daily quality controls as part of a quality process.

### Safety and adherence

The number, nature and incidence of all grade-3 or -4 (on the ANRS severity scale of adverse effects) clinical and biological events (transaminases, bilirubin, creatinine, haemoglobin, platelets, leukocytes and neutrophils) occurring or worsening after Week (W) 0 were measured^19^; along with the number, nature and incidence of grade-3 or -4 clinical and biological events attributable to trial treatments occurring or worsening after W0.

A questionnaire was also administered at each follow-up visit to collect, among other information, self-reported adherence to trial treatment. Pill-count based adherence was estimated using the percentage of treatment taken (total number of tablets given minus total number of tablets returned) divided by the total number of tablets prescribed for each drug. Moreover, adherence items in the questionnaire were used to estimate adherence by classifying patients as having high, medium or low adherence to each treatment^20^.

### Ethics

The study protocol was approved by each country’s national ethics committee (Senegal: 29 May 2015, no 98; Cameroon: 11 May 2015, no 2015/05/589; Côte d’Ivoire: 7 July 2015, no 033/MSLS/CNER-dkn) and by the Ethics Committee of Ile de France XI, France (16 December 2014, CPP Ile de France XI, no 14085). The study was registered on clinicaltrials.gov (NCT02405013) on 01/04/2015. Each participant provided an informed consent form.

All methods were performed in accordance with the relevant guidelines and regulations.

### Statistical analysis

Sample size was based on Fleming/A Hern’s design. With a unilateral type-I error of 5% and 80% power, 37 patients per genotype group were required, to test the null hypothesis of < 50% efficacy against the alternative hypothesis of at least 70% efficacy (as reported in trials based on interferon). Considering a 10% lost-to-follow-up rate, sample size was increased to 40 in each genotype subgroup to be able to conduct a complete case analysis.

The primary endpoint was a sustained virological response 12 weeks after EOT (SVR12). Secondary endpoints included SVR 24 weeks after treatment, safety during treatment, adherence and retention in treatment, evolution of HIV markers among HIV-HCV co-infected patients and SVR 12 weeks after end of retreatment. The primary efficacy analysis was performed on an intention-to-treat (ITT) basis. In each genotype group, the proportion of patients reaching the primary endpoint and the corresponding 90% confidence interval (CI) were calculated, and the lower boundary was compared with 50%. Given the low rate of virological failure, association between possible predictors (HCV genotype, APRI, HIV status and adherence score) were studied by univariate graphical description with 95% CI.

Statistical analysis was performed with SAS software version 9.4.

## Results

**Figure 1:**
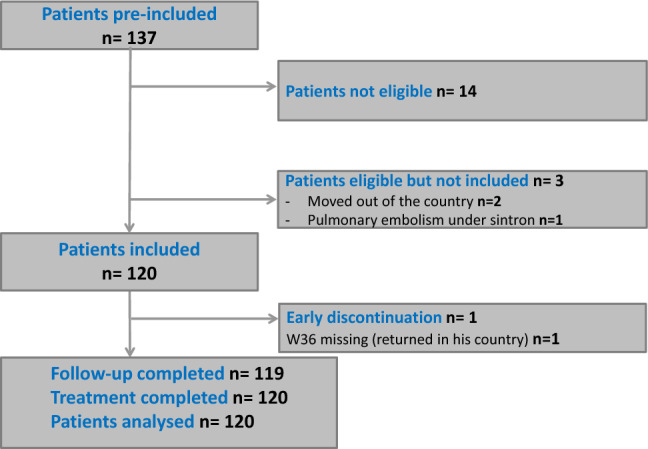
Study flow diagram.

Between November 9, 2015 and March 1, 2017, 137 patients were screened for eligibility, 14 were considered ineligible whereas three potentially eligible individuals were not included because of migration to another country (N = 2) or ongoing anticoagulant treatment for pulmonary embolism (N = 1). Therefore, 120 patients were enrolled (40 for each genotype 1, 2 and 4). The follow-up of first-line treatment was completed on November 2017, whereas the patients failing 1st line were follow-up until November 2019 (Fig. 1). Table 1 presents patient distribution by country and genotype.

**Table 1:** Baseline (W0) characteristics by HCV genotype (n = 120).

	Genotype 1	Genotype 2	Genotype 4	Overall
	n = 40 n (%) or Median [IQR]	n = 40 n (%) or Median [IQR]	n = 40 n (%) or Median [IQR]	n = 120 n (%) Median [IQR]
Male gender	21 (53)	25 (63)	19 (48)	65 (54)
Age (year)	59 [51–64]	52 [41–60]	61 [55–63]	58 [49–63]
Country of enrolment				
Cote d’Ivoire	27 (68)	18 (45)	0 (0)	45 (38)
Cameroon	4 (10)	13 (33)	36 (90)	53 (44)
Senegal	9 (22)	9 (22)	4 (10)	22 (18)
BMI (kg/m^2^)	23.6 [19.9–25.9]	24.3 [21.9–27.6]	28.0 [24.3–31.2]	24.9 [22.0–28.4]
Alcohol, Yes, < once a day	2 (5)	8 (20)	11 (28)	21 (18)
HIV infection	8 (20)	12 (30)	16 (40)	36 (30)
Liver fibrosis (APRI low > 1)	9 (23)	10 (25)	10 (25)	29 (24)
Cirrhosis (APRI high > 2)	3 (8)	6 (15)	5 (13)	14 (12)
logHCV-RNA (IU/mL) at W0	5.8 [5.2–6.3]	6.0 [5.3–6.4]	6.4 [5.8–6.7]	6.0 [5.5–6.6]
ALT* (IU/L) at W0	50 [36–71]	46 [26–89]	49 [39–80]	47 [36–78]
ALT grade at W0				
0	23 (58)	26 (65)	25 (63)	74 (62)
1.25–2.50 × ULN^¥^	15 (38)	7 (18)	10 (25)	32 (27)
> 2.50–5.00 × ULN	2 (5)	5 (13)	4 (10)	11 (9)
> 5.00–10.00 × ULN	0	2 (5)	1 (3)	3 (3)
Creatinine (mg/L) at W0	7 [6–10]	9 [8–10]	8 [7–10]	8 [7–10]
Hemoglobin (g/dL) at W0	13 [12–14]	14 [13–15]	14 [13, 14]	14 [12–14]

*Alanine aminotransferase.

^¥^Under Limit of Normal.

### Participants’ main characteristics at baseline

Approximately half (54%) of the trial participants were men, median [interquartile range (IQR)] age was 58 [49–63] years, 30% were co-infected with HIV (CD4: 624 [424–844] cells/mm^3^, all with plasma HIV-RNA < 200 copies/mL). APRI was > 2 in 12% of participants, indicating the probable cirrhotic state. The median [IQR] HCV viral load was 6.0 [5.5–6.6] log_10_ copies/mL; 3% had ALT > 5 ULN and the median [IQR] haemoglobin level was 14 [12–14] g/dL (Table 1).

### SVR12 and treatment failure

**Figure 2:**
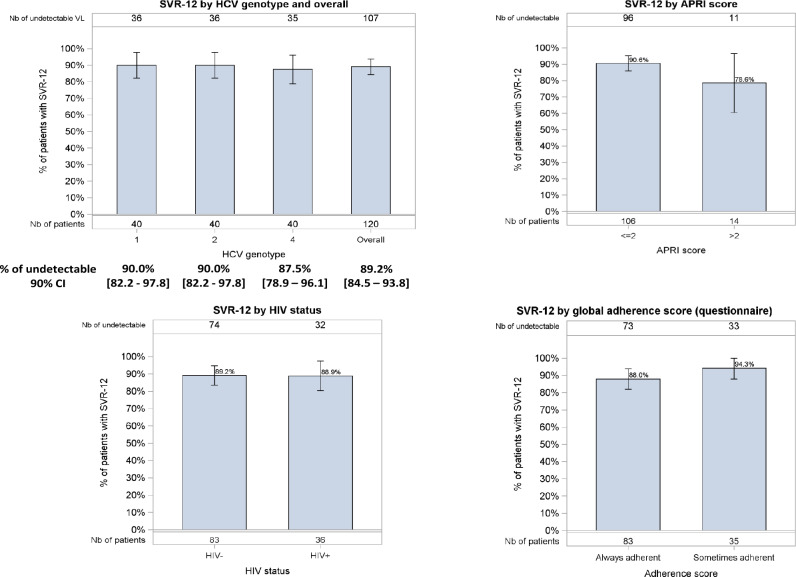
Rates of sustained virological response at Week 24 (SVR-12) according to patients’ characteristics.

SVR12 was assessed in all patients and was reached in 89% (107/120) of the participants and did not differ according to the HCV genotype or HIV coinfection. Figure 2 shows the distribution of SVR12 by HCV genotype, APRI, HIV co-infection and adherence score. The low rate of virological failures only allowed for a univariable analysis [with the genotype, APRI, HIV status and adherence as explanatory variables]. None of these factors was found to be associated with SVR12.

**Figure 3:**
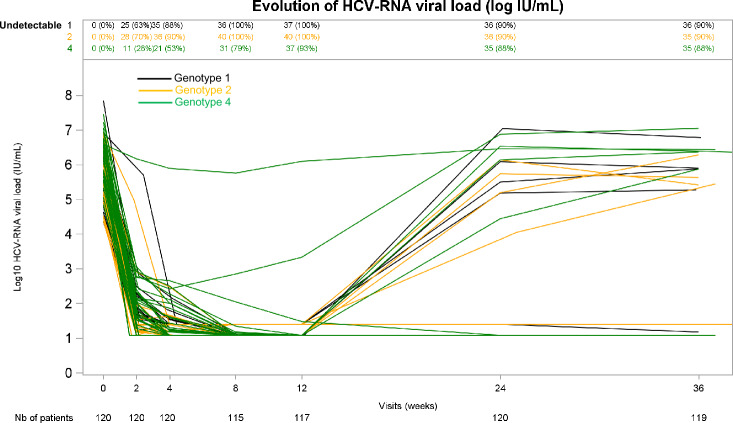
Evolution of plasma HCV-RNA level (log IU/mL).

At W8, the percentage of individuals with undetectable HCV viral load was 100% for all genotypes except for GT-4 (88%) (p = 0.0002). The evolution of HCV viral load over time in the study population is reported in Fig. 3.

Viral load was assessed at W36 in all patients except one who was lost-to-follow-up (detectable at W24). SVR24 (W36) was reached in 106/119 patients (89%), and the rate of SVR24 was similar across genotypes.

Thirteen patients (11%) did not respond to the first-line DAA, six of them were cirrhotic at baseline. These patients were all retreated with SOF/VEL/VOX, and all achieved SVR at W12 after retreatment initiation.

### Safety

We recorded 3 drug-induced adverse events (1 hypertensive surge, 2 high blood pressures) and 11 other grade-3 or -4 adverse events not considered associated with HCV treatment (1 malaria, 1 pulmonary embolism, 2 neutropenia, 1 leucopenia, 1 lower back pain, 1 left atrial pain, 1 hepatic cytolysis, 1 shoulder trauma, 1 hyperalgesic osteoarthritis, 1 hand paralysis). One patient had an AIDS event, an oesophageal candidosis started 5 days after W0. No patient experienced a grade-2–4 anaemia, including those who received RBV.

### Treatment adherence

All patients except one (with HCV viral load undetectable at W24 and W36) completed the DAA course.

At W12, 90% and 89% of patients with GT-1 and -4 respectively reported high adherence to SOF + LDV (GT-1 and -4) while for patients with GT-2, high adherence to SOF and RBV was found in 95% and 79% of patients, respectively. For SOF + LDV, median [IQR] pill count ratio was 100% [94–100% and 83% [71–98%], in GT-1 and -4 respectively. For patients with GT-2, median [IQR] pill count ratio was 94% [79–100%] for SOF and 97% [92–100%] for RBV.

All patients were retained in treatment except one who discontinued follow-up early to return to his native country.

## Discussion

This study reports the efficacy rates and safety of sfosbuvir-based all oral regimen as first-line treatment and retreatment, in CHC people in three sub-Saharan African countries (Cameroon, Cote d’Ivoire, Senegal). First, our study found high SVR rates after 12 weeks, comparable to SVR rates obtained in other African regions^8,21^ or among migrants from SSA^13^. In those studies, such high SVR rates were shown to be associated with a high self-reported adherence rates to DAAs. Secondly, slightly (but not significant) reduced effectiveness was observed for patients with GT-4 or cirrhosis; however, retreatment with a different combination resulted in SVR at Week 12 of the second-line treatment. Finally, the third and equally important result is that this study confirms that HCV cure is possible in these settings when HCV care and treatment are free for all patients and when it is possible to offer a second-line treatment in case of virological failure with the first-line treatment. The latter result indirectly underlines how these findings advocate for a universal and free model of HCV care for all patients living with chronic HCV infection in SSA. Such a model of care can be implemented and scaled up in a test-and-treat strategy to achieve the World Health Organization HCV elimination goal by 2030.

Studies conducted in SSA remain sparse. Our results are corroborated by three other studies conducted in different SSA settings. The first, in Rwanda, has shown an overall 12-week SVR rate of 87% after a SOF-LDV combined therapy. The study showed lower SVR12 rates in patients with GT-4r^9^, a result that may not be valid for West and Central Africa where HCV characteristics (i.e., genotypes, sub-genotypes, co-infections) and environment differ greatly from Eastern Africa. The second study was performed in six specialized gastroenterology clinics in Yaounde (Cameroon). Patients received 12 or 24 weeks of a fixed dose of branded SOF-LDV with or without ribavirin, depending on the stage of liver disease and HCV genotype. The overall SVR12 rate was 96.2%, with no difference according to HCV genotype^21^. A very recent multicountry study (including South Africa) was conducted and assessed the effectiveness of an HCV cure simplified model based on minimal monitoring, i.e., no genotyping, dispensing the entire treatment course at enrolment, no scheduled visits or laboratory monitoring and two points of remote contacts during treatment^22^. In this phase 4 study, participants received a fixed-dose combination of oral SOF and VEL once daily for 12 weeks; results showed 95% SVR12 rates and comparable high adherence rates. This study confirmed the feasibility and effectiveness of this alternative (and free) model of care. Moreover, the multicountry study confirms that access to DAAs facilitates general retention in HCV care, as already demonstrated in a different context for underserved populations^23^.

Concerning lack of SVR12 after first-line DAA treatment failure, adherence cannot explain all cases of failure, since there is an inter-individual variability in the treatment of viral hepatitis C due to host genetics^24,25^. However, though side effects are limited for DAAs and generally do not affect adherence^26^, the surveillance and management of incident adverse events whether or not attributable to HCV treatment, remain central issues. Viral failure may also be associated with new HCV subtypes for which a decrease of DAA efficacy has been reported in Rwanda for example^9^. However, in implementation programmes, genotyping is not compatible with limited resources and pangenotypic regimens are suggested as more effective for HCV elimination. Though new subtypes continue to be documented^27^, there is still a considerable lack of data about new sub-genotypes spread in SSA^28^.

It is worth noting that the only adverse events validated as attributable to antiviral treatment were hypertension events, as also found in the Rwandan study (32% of adverse events)^9^*.* Across all WHO regions, including the Americas, Africa has the highest prevalence of hypertension, where 46% of the entire population over 25 years of age is estimated to be hypertensive^29^*.* Yet for over 20 years, in SSA, the prevention, detection, management and control of hypertension with multifaceted causes has been considered a high priority^29–31^. Unfortunately, frequent reports indicate that these measures remain haphazard and insufficient^31^.

While our study effectively demonstrates the feasibility, efficacy, excellent adherence, and good tolerance within a structurally constrained context, its findings retain validity for the studied population and align with the proposed model of care, primarily centered on HCV response monitoring and patient educational sessions. While frequent viral load monitoring was implemented due to the study’s inception during a period of uncertain treatment efficacy, such stringent monitoring protocols are now considered obsolete. There is no scientific basis to replicate this level of monitoring in future studies, especially those focused on implementation. Instead, a singular assessment at the conclusion of HCV treatment suffices. This primarily indicates that for replicating our results within a scale-up framework, implementation models should prioritize patient education, potentially facilitated by community members, to enhance efforts towards HCV elimination.

Indeed, the scale-up of any model of HCV cure and elimination in these settings encounters several challenges and unmet needs^7^. First, effective access to treatment and laboratory monitoring remains a major barrier as the costs of the drugs—even generics—and biological tests are still not affordable for the majority of populations affected by chronic HCV infection. Next, despite the ongoing spread of point-of-care test in some African countries, expanding HCV testing and access to DAAs to reach HCV elimination is challenging because the increase in expenditures to ensure effective case finding and HCV cure is too onerous to governments or potentially so to individuals and families^32,33^. Concerning access to DAA, agreements with the two DAA drug license owners (Gilead for sofosbuvir, ledipasvir and velpatasvir, and Bristol-Myers Squibb for daclatasvir) allow for the production and sale of generic DAAs in 100 LMICs^34,35^. Using this study’s data, Boyer et al. has recently shown that at generic cost, sofosbuvir/daclatasvir provided the best value for money (incremental cost-effectiveness ratios (ICERs) range: US$139–216/quality-adjusted life years according to country) due to significantly lower lifetime costs^35^. Funding interventions based on such low cost-effectiveness tools (CETs) would bring additional health benefits at population level since their cost/quality-adjusted life years is lower than that of other funded interventions like antiretrovirals for HIV^36^. Concerning DAA availability, while the importation and manufacture of generic DAA is now possible, there are still two important regulatory steps necessary before DAAs—generic or originator—can be sold in a country: WHO pre-qualification and market authorization by national authorities. These two steps are urgent, considering that a recent modelling study on the economic and health effect of voluntary licensing for medicines for HIV and HCV in LMICs^37^ has clearly shown that for daclatasvir-based HCV treatments, the cumulative effect from 2015 to 2026 would result in an additional uptake of approximately 400,000 patients, 4000 deaths averted and on average 107,593 saved with the licence compared with the counterfactual scenario.

As mentioned above, scale-up of innovative diagnostic and monitoring tools developed to improve screening and liver assessment at a low cost are needed as well as devices adapted to the local African context to rapidly quantify viral load (i.e., SVR12) at a lower cost^7^. Moreover, implementation research approaches should be further promoted to better detail how to introduce innovative testing strategies and point-of-care, how to engage people in care after diagnosis and how to monitor clinical response and adverse effects in experienced patients, even those living in decentralised areas^38^. The case of Egypt is emblematic, as this country represents a major field experience to policy makers internationally, on how the hepatitis C cascade can be implemented. It also offers a pragmatic guide on managing the difficulties encountered with such struggling testing and treatment programmes^39^.

In these settings^39^ as in European countries, one major barrier to successful models of HCV elimination is related to stigmatizing attitudes towards people living with HCV. These generate self-stigma, which impedes sharing serostatus with family members and friends and severely affects engagement in and continuity of HCV care. Improving patient-provider communication about barriers to full patient engagement in care after diagnosis and fighting stigma in health settings are major components of a successful HCV-elimination strategy^14^. In addition, the COVID-19 pandemic has partially slowed down efforts to implement HCV-treatment programmes in LMICs^40^ as health policies and resources have been diverted by the pandemic. Due to the COVID-19 economic effects on the health system, free HCV care seems to be an unattainable objective unless international funding mechanisms are promoted to reduce the increasing burden associated with viral hepatitis.

Some limitations and strengths need to be acknowledged for our study. First, it was designed before the dissemination of recommendations about suggesting adopting pan-genotyping treatments as first-line treatments. Indeed, the study was funded in 2015, implemented between 2015 and 2019 when considering retreatment of patients failing first-line therapy. Then the Covid19 pandemic has considerably slowed down the collection of remaining data as a consequence of successive periods of lockdowns in each country of the trial. Secondly, the study was not conducted to face the challenges of remote or rural populations needing HCV care, and further studies about how to deliver decentralised testing and care are needed^41^. Nevertheless, the study allowed full gratuity of first- and second-line treatment and HCV clinical follow-up. This gratuity was a main strength and key to treatment success, as is already the case for HIV treatment in SSA. Another important strength is that the study also assessed self-reported adherence, which can contribute to understanding the risk of failure to first-line DAAs. Another strength was the opportunity to create a network for HCV screening and care in the areas where it was conducted. This network now has the potential to reinforce collaboration between governments and ministries of health to improve the management of viral hepatitis in Africa, strengthen the partnership with NGOs involved in viral hepatitis control in Africa and provide international research agencies and WHO with new data on HCV in Africa, which are currently lacking, and thus help to establish recommendations adapted to the African context.

## Conclusion

HCV treatment is acceptable, safe and effective under this model of care. Implementation research adapting this model by scaling-up point-of-care for HCV testing and SVR assessment and community involvement for patient education is now urgent for reaching HCV elimination in Sub-Saharan Africa.

## Data Availability

The datasets generated during and/or analysed during the current study are not publicly available, but are available upon reasonable request. Person contact: Corine Chazallon: corine.chazallon@u-bordeaux.fr.
